# Crystal structure of (diethyl ether-κ*O*)[5,10,15,20-tetra­kis­(2-iso­thio­cyanato­phen­yl)porphyrinato-κ^4^ *N*]zinc diethyl ether solvate

**DOI:** 10.1107/S2056989018014238

**Published:** 2018-10-19

**Authors:** Lisa Leben, Eike Schaub, Christian Näther, Rainer Herges

**Affiliations:** a Institut für Organische Chemie, Universität Kiel, Otto-Hahn-Platz 4, 24118, Kiel, Germany; b Institut für Anorganische Chemie, Universität Kiel, Otto-Hahn-Platz 6/7, 24118, Kiel, Germany

**Keywords:** crystal structure, picket fence porphyrin, zinc(II) porphyrin, atropisomer, iso­thio­cyanate

## Abstract

The crystal structure of the title compound, [Zn(C_48_H_24_N_8_S_4_)(C_4_H_10_O)]·C_4_H_10_O, consists of discrete porphyrin complexes that are located on a twofold rotation axis. The Zn^II^ cation is fivefold coordinated by four N atoms of the porphyrin moiety and one O atom of a diethyl ether mol­ecule in a slightly distorted square-pyramidal environment with the diethyl ether mol­ecule in the apical position. The porphyrin backbone is nearly planar with the metal cation slightly shifted out of the plane towards the coordinating diethyl ether mol­ecule. All four iso­thio­cyanato groups of the phenyl substituents at the *meso*-positions face the same side of the porphyrin, as is characteristic for picket fence porphyrins. In the crystal structure, the discrete porphyrin complexes are arranged in such a way that cavities are formed in which additional diethyl ether solvate mol­ecules are located around a twofold rotation axis. The O atom of the solvent mol­ecule is not positioned exactly on the twofold rotation axis, thus making the whole mol­ecule equally disordered over two symmetry-related positions.

## Abstract 2

The synthesis and crystal structure of 5,10,15,20-tetra­kis *α,α,α,α* 2-iso­thio­cyanato­phenyl zinc(II) porphyrin are reported. The crystal structure consists of discrete porphyrin complexes that are located on a twofold rotation axis with the Zn^II^ cation in a square-pyramidal coordination environment defined by the porphyrin N atoms at the basal sites and a diethyl ether mol­ecule at the apical site.

## Chemical context   

**Figure 1:**
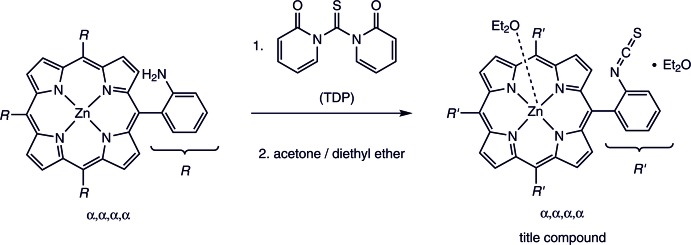
Reaction scheme for the synthesis of the title compound.

Iso­thio­cyanates serve as versatile starting materials for a variety of functional groups (Batey & Powell, 2000 ▸; Ding *et al.*, 2011 ▸; Serra *et al.*, 2014 ▸; Guo *et al.*, 2010 ▸; Shin *et al.*, 2000 ▸; Kosurkar *et al.*, 2014 ▸; Alizadeh *et al.*, 2016 ▸; Rao *et al.*, 2015 ▸). Included in porphyrin scaffolds, iso­thio­cyanates may serve as precursors for the synthesis of tetra­topic ligands with fourfold symmetry. In the case where all four *ortho*-substituents of the *meso*-phenyl groups face the same side of the porphyrin plane, these porphyrins are denominated picket fence porphyrins. These compounds are widely used as model compounds for hemoproteins (Collman *et al.*, 1975 ▸; Tabushi *et al.*, 1985 ▸; Schappacher *et al.*, 1989 ▸). With a bulky *ortho*-substituent and Zn^II^ as the central metal cation, the rotational barriers are sufficiently high to isolate the different atropisomers (Freitag & Whitten, 1983 ▸). A variety of picket fence porphyrins has been reported (Collman *et al.*, 1975 ▸; Mansour *et al.*, 2017 ▸; Cormode *et al.*, 2006 ▸; Le Maux *et al.*, 1993 ▸; Wuenschell *et al.*, 1992 ▸). In most cases, amides are used as functional groups in the *ortho*-positions of the *meso*-phenyl groups, which hampers further functionalization. The title compound now opens new avenues for the synthesis of functionalized picket fence porphyrins and is a promising starting material for the design of anion binding ligands. The title compound can be obtained in one step using a method reported by Jha *et al.* (Fig. 1 ▸), starting from the all-*α* isomer of the amino derivative we have published previously (Jha *et al.*, 2007 ▸; Leben *et al.*, 2018 ▸). It is important to note that the reaction has to be carried out at 273 K, because at room temperature a mixture of the atrop­isomers is obtained. After dissolving the tetra­kis­(iso­thio­cyanato­phen­yl) porphyrin in acetone and precipitating with diethyl ether, single crystals were obtained, which were characterized by single crystal X-ray diffraction.
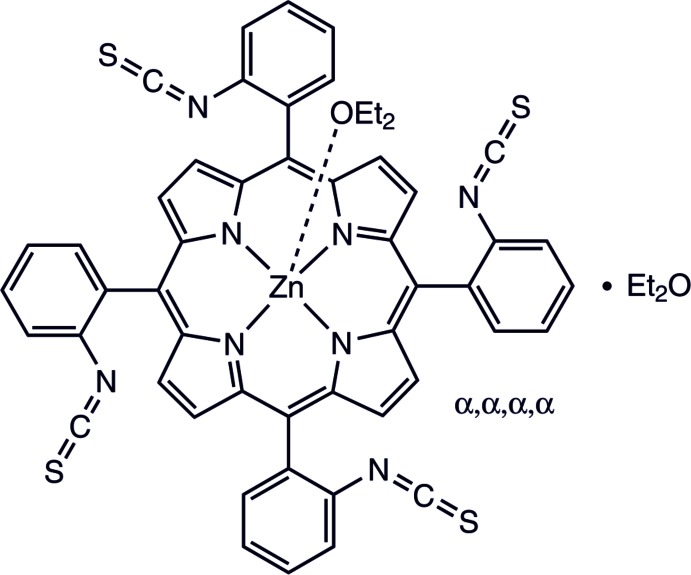


## Structural commentary   

**Figure 2:**
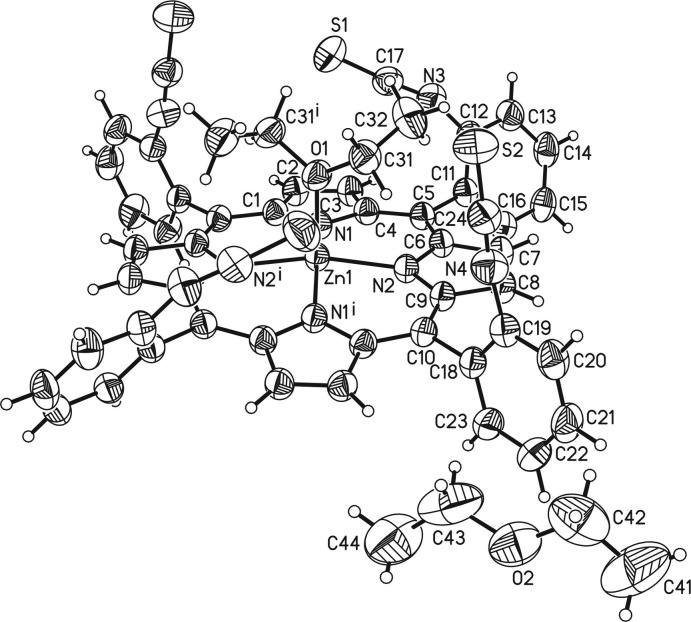
The mol­ecular entities of the title compound with the atom labelling and displacement ellipsoids drawn at the 50% probability level. Only one orientation of the disordered diethyl ether solvent is given. [Symmetry code: (i) −*x* + 2, *y*, −*z* + 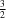.]

**Figure 3:**
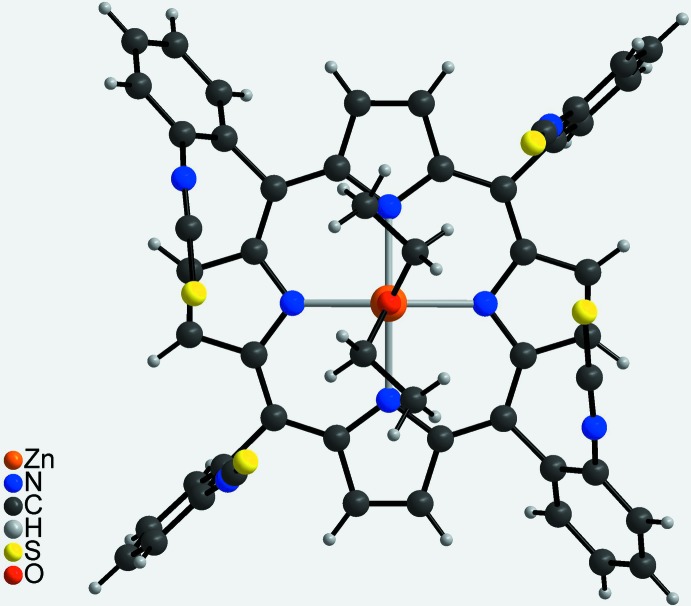
Mol­ecular structure of the discrete complex in a view onto the porphyrin plane.

**Figure 4:**
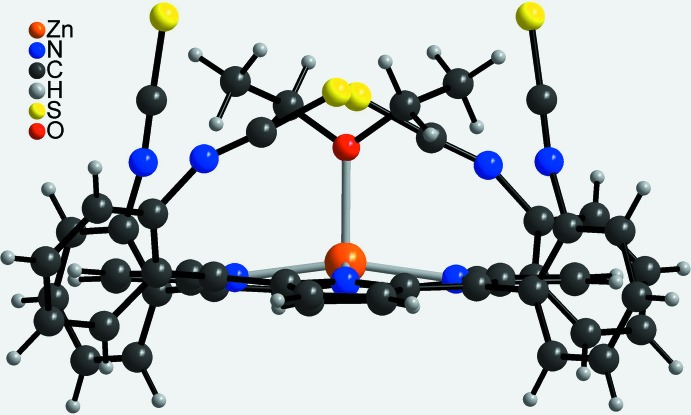
Mol­ecular structure of the discrete complex in a view parallel to the porphyrin plane.

The asymmetric unit of the title compound, Zn(C_48_H_24_N_8_S_4_)(C_4_H_10_O)·C_4_H_10_O, comprises one Zn^II^ cation, one half of the porphyrin mol­ecule and one half of a coordinating diethyl ether mol­ecule as well as one half of a diethyl ether solvate mol­ecule. The complex porphyrin mol­ecule and the coordinating diethyl ether mol­ecule are located on a twofold rotation axis whereas the solvent diethyl ether mol­ecule is in a general position and is equally disordered around a twofold rotation axis (Fig. 2 ▸). The four iso­thio­cyanate substituents of the phenyl groups at the *meso*-positions point to the same side of the porphyrin moiety, which proves that the tetra-*α* isomer has formed. The porphyrin plane is close to planar with a maximum deviation from the mean plane of 0.276 (3) Å. The phenyl rings are rotated out of the porphyrin plane by 63.16 (5) and 82.06 (6)°. The Zn^II^ cation is fivefold coordinated by the four N atoms of the porphyrin mol­ecule in the basal positions and by one O atom of a diethyl ether mol­ecule in the apical position, leading to a distorted square-pyramidal coordination environment (Table 1 ▸, Fig. 3 ▸). The Zn—N distances of 2.0622 (13) and 2.0684 (14) Å and the Zn—O distance of 2.1352 (19) Å are in characteristic ranges. The angles around the Zn^II^ cation range from 88.54 (6) to 99.69 (4)° for the basal N_4_ plane and from 160.61 (8) to 164.44 (8)° involving the apical O atom, demonstrating that the square pyramid is slightly distorted (Table 1 ▸). The Zn^II^ cation is located 0.4052 (9) Å out of the mean porphyrin plane and is shifted towards the coordinating diethyl ether mol­ecule (Fig. 4 ▸).

## Supra­molecular features   

**Figure 5:**
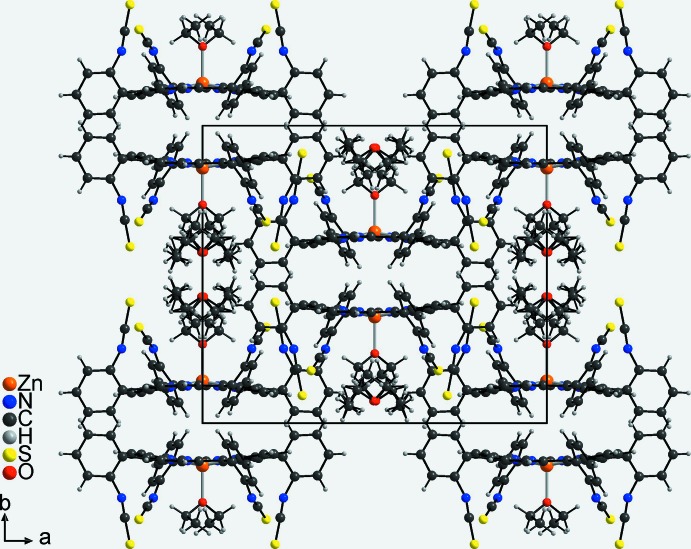
Crystal structure of the title compound viewed along [001].

In the crystal structure of the title compound, each two discrete complexes form centrosymmetric pairs with the coordinating diethyl ether mol­ecules pointing in opposite directions (Fig. 5 ▸). The complexes are arranged into columns along [001]. This arrangement leads to the formation of cavities between two neighbouring coordinating diethyl ether mol­ecules, in which the disordered diethyl ether solvate mol­ecules are embedded (Fig. 5 ▸). There are no notable inter­molecular inter­actions between the mol­ecular moieties in the crystal structure.

## Database survey   

The synthesis of the metal-free oxygen derivative 5,10,15,20-tetra­kis *α,α,α,α* 2-iso­cyanato­phenyl porphyrin has been known for several years (Collman *et al.*, 1998 ▸). However, the crystal structure of this compound has not yet been reported. A CSD database search (Version 5.39; Groom *et al.*, 2016 ▸) revealed the crystal structures of several metal porphyrins with iso­thio­cyanate entities as axial ligands (Dhifet *et al.*, 2010 ▸; Scheidt *et al.*, 1982 ▸; Ezzayani *et al.*, 2014 ▸; Denden *et al.*, 2015 ▸). In addition, the crystal structure of a *para*-iso­thio­cyanato­phenyl porphyrin has been reported (Sibrian-Vazquez *et al.*, 2005 ▸).

## Synthesis and crystallization   

The metal-free all-*α* isomer of 2-amino­phenyl porphyrin was synthesized according to reported procedures (Collman *et al.*, 1975 ▸; Lindsey, 1980 ▸). Metallation followed standard metallation conditions as reported previously (Strohmeier *et al.*, 1997 ▸; Leben *et al.*, 2018 ▸). For the introduction of the iso­thio­cyanato groups, a modified synthesis was used (Jha *et al.*, 2007 ▸). 5,10,15,20-Tetra­kis(*α,α,α,α* 2-amino­phen­yl)zinc(II) porphyrin (150 mg, 203 µmol) was dissolved in 30 ml of di­chloro­methane and cooled to 273 K. 1,1′-Thio­carbonyldi-2,2′-pyridone (TDP, 377 mg, 1.62 mmol) was added and the mixture stirred for 50 minutes at 273 K. Removing the solvent and filtration over silica gel (cyclo­hexa­ne/ethyl acetate, *v*:*v* = 1:1) gave the title compound in qu­anti­tative yield. For crystallization, a small amount was dissolved in acetone and crystallized by adding diethyl ether.

^1^H NMR (500 MHz, CDCl_3_, 300 K): *δ* = 8.80 (*s*, 8H, H-*β*), 8.21 (*dd*, ^3^ *J* = 7.5 Hz, ^4^ *J* = 1.2 Hz, 4H, H-6), 7.78 (*dt*, ^3^ *J* = 7.9 Hz, ^4^ *J* = 1.5 Hz, 4H, H-4), 7.68 (*dt*, ^3^ *J* = 7.6 Hz, ^4^ *J* = 1.3 Hz, 4H, H-5), 7.61 (*dd*, ^3^ *J* = 8.2 Hz, ^4^ *J* = 1.0 Hz, 4H, H-3) ppm. ^13^C NMR (125 MHz, CDCl_3_, 300 K): *δ* = 149.9 (C-*α*), 141.0 (C1), 134.8 (C6), 134.5 (C2), 131.6 (C-*β*), 129.3 (C4), 125.7 (C5), 124.4 (C3), 115.7 (C-*meso*) ppm. EI–MS (70 eV): *m*/*z* (%) = 904.1 (100) [*M*]^+^.

## Refinement   

Crystal data, data collection and structure refinement details are summarized in Table 2 ▸. The C—H hydrogen atoms were positioned with idealized geometries (C—H = 0.95–0.99 Å; methyl H atoms of the coordinating diethyl ether mol­ecule were allowed to rotate but not to tip) and were refined with *U*_iso_(H) = 1.2*U*_eq_(C) (1.5 for methyl H atoms) using a riding model. The O atom of the diethyl ether solvate mol­ecule is not located exactly on the twofold rotation axis and thus the complete mol­ecule is equally disordered over two sets of sites because of symmetry. Therefore for each atom the occupancy was set to 0.5, and atoms were treated with SADI and SIMU commands (Sheldrick, 2015*b* ▸) to achieve similar displacement ellipsoids.

## Supplementary Material

Crystal structure: contains datablock(s) I. DOI: 10.1107/S2056989018014238/wm5466sup1.cif

Structure factors: contains datablock(s) I. DOI: 10.1107/S2056989018014238/wm5466Isup2.hkl

CCDC reference: 1872076

Additional supporting information:  crystallographic information; 3D view; checkCIF report

## Figures and Tables

**Table 1:** Selected geometric parameters (Å, °)

Zn1—N2	2.0622 (13)	Zn1—N1	2.0685 (14)
Zn1—N2^i^	2.0622 (13)	Zn1—O1	2.1352 (19)
Zn1—N1^i^	2.0684 (14)		
			
N2—Zn1—N2^i^	164.44 (8)	N1^i^—Zn1—N1	160.61 (8)
N2—Zn1—N1^i^	88.85 (6)	N2^i^—Zn1—O1	97.78 (4)
N2^i^—Zn1—N1^i^	88.54 (6)	N1^i^—Zn1—O1	99.69 (4)
N2—Zn1—N1	88.54 (6)	N1—Zn1—O1	99.69 (4)
N2^i^—Zn1—N1	88.85 (6)		

Symmetry code: (i) 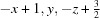.

**Table 2:** Experimental details

Crystal data	
Chemical formula	[Zn(C_48_H_24_N_8_S_4_)(C_4_H_10_O)]·C_4_H_10_O
*M*_r_	1054.60
Crystal system, space group	Monoclinic, *C*2/*c*
Temperature (K)	200
*a*, *b*, *c* (Å)	19.8830 (4), 17.1781 (3), 14.8684 (3)
β (°)	91.667 (1)
*V* (Å^3^)	5076.18 (17)
*Z*	4
Radiation type	Mo *K*α
μ (mm^−1^)	0.70
Crystal size (mm)	0.14 × 0.11 × 0.07

Data collection	
Diffractometer	Stoe IPDS2
Absorption correction	Numerical (*X-RED* and *X-SHAPE*; Stoe, 2008 ▸)
*T*_min_, *T*_max_	0.807, 0.951
No. of measured, independent and observed [*I* > 2σ(*I*)] reflections	39705, 5530, 5042
*R*_int_	0.039
(sin θ/λ)_max_ (Å^−1^)	0.639

Refinement	
*R*[*F* ^2^ > 2σ(*F* ^2^)], *wR*(*F* ^2^), *S*	0.036, 0.103, 1.05
No. of reflections	5530
No. of parameters	346
No. of restraints	26
H-atom treatment	H-atom parameters constrained
Δρ_max_, Δρ_min_ (e Å^−3^)	0.39, −0.35

Computer programs: *X-AREA* (Stoe, 2008 ▸), *SHELXT* (Sheldrick, 2015*a* ▸), *SHELXL2014* (Sheldrick, 2015*b* ▸), *XP* (Sheldrick, 2008 ▸), *DIAMOND* (Brandenburg, 2014 ▸) and *publCIF* (Westrip, 2010 ▸).

## supplementary crystallographic information

### Crystal data

**Table:**

[Zn(C_48_H_24_N_8_S_4_)(C_4_H_10_O)]·C_4_H_10_O	*F*(000) = 2184
*M**_r_* = 1054.60	*D*_x_ = 1.380 Mg m^−^^3^
Monoclinic, *C*2/*c*	Mo *K*α radiation, λ = 0.71073 Å
*a* = 19.8830 (4) Å	Cell parameters from 39705 reflections
*b* = 17.1781 (3) Å	θ = 1.6–27.0°
*c* = 14.8684 (3) Å	µ = 0.70 mm^−^^1^
β = 91.667 (1)°	*T* = 200 K
*V* = 5076.18 (17) Å^3^	Block, red
*Z* = 4	0.14 × 0.11 × 0.07 mm

### Data collection

**Table:**

Stoe IPDS-2 diffractometer	5042 reflections with *I* > 2σ(*I*)
ω scans	*R*_int_ = 0.039
Absorption correction: numerical (X-Red and X-Shape; Stoe, 2008)	θ_max_ = 27.0°, θ_min_ = 1.6°
*T*_min_ = 0.807, *T*_max_ = 0.951	*h* = −25→25
39705 measured reflections	*k* = −21→21
5530 independent reflections	*l* = −18→18

### Refinement

**Table:**

Refinement on *F*^2^	Hydrogen site location: mixed
Least-squares matrix: full	H-atom parameters constrained
*R*[*F*^2^ > 2σ(*F*^2^)] = 0.036	*w* = 1/[σ^2^(*F*_o_^2^) + (0.0603*P*)^2^ + 2.7141*P*] where *P* = (*F*_o_^2^ + 2*F*_c_^2^)/3
*wR*(*F*^2^) = 0.103	(Δ/σ)_max_ = 0.001
*S* = 1.05	Δρ_max_ = 0.39 e Å^−^^3^
5530 reflections	Δρ_min_ = −0.35 e Å^−^^3^
346 parameters	Extinction correction: SHELXL, Fc^*^=kFc[1+0.001xFc^2^λ^3^/sin(2θ)]^-1/4^
26 restraints	Extinction coefficient: 0.0011 (2)

### Special details

**Table:**

Geometry. All esds (except the esd in the dihedral angle between two l.s. planes) are estimated using the full covariance matrix. The cell esds are taken into account individually in the estimation of esds in distances, angles and torsion angles; correlations between esds in cell parameters are only used when they are defined by crystal symmetry. An approximate (isotropic) treatment of cell esds is used for estimating esds involving l.s. planes.

### Fractional atomic coordinates and isotropic or equivalent isotropic displacement parameters (Å^2^)

**Table:**

	*x*	*y*	*z*	*U*_iso_*/*U*_eq_	Occ. (<1)
Zn1	0.5000	0.64166 (2)	0.7500	0.03835 (10)	
N1	0.40708 (7)	0.62138 (9)	0.68826 (9)	0.0400 (3)	
N2	0.54332 (7)	0.62541 (9)	0.62706 (9)	0.0392 (3)	
C1	0.34674 (8)	0.61377 (10)	0.72962 (11)	0.0405 (3)	
C2	0.29355 (9)	0.60302 (11)	0.66268 (12)	0.0460 (4)	
H2	0.2471	0.5962	0.6736	0.055*	
C3	0.32240 (9)	0.60449 (12)	0.58175 (12)	0.0464 (4)	
H3	0.3001	0.5983	0.5248	0.056*	
C4	0.39333 (8)	0.61718 (10)	0.59772 (11)	0.0407 (3)	
C5	0.44062 (9)	0.62460 (10)	0.53022 (11)	0.0408 (3)	
C6	0.51067 (9)	0.62744 (10)	0.54465 (11)	0.0405 (3)	
C7	0.55953 (9)	0.62818 (12)	0.47508 (12)	0.0472 (4)	
H7	0.5503	0.6310	0.4121	0.057*	
C8	0.62118 (9)	0.62418 (12)	0.51605 (12)	0.0473 (4)	
H8	0.6632	0.6226	0.4873	0.057*	
C9	0.61077 (8)	0.62273 (10)	0.61146 (11)	0.0407 (3)	
C10	0.66219 (8)	0.61682 (10)	0.67757 (11)	0.0406 (3)	
C11	0.41493 (9)	0.62820 (11)	0.43487 (11)	0.0423 (4)	
C12	0.37604 (9)	0.69080 (12)	0.40320 (12)	0.0479 (4)	
C13	0.35525 (11)	0.69580 (14)	0.31334 (13)	0.0578 (5)	
H13	0.3294	0.7392	0.2928	0.069*	
C14	0.37198 (10)	0.63815 (14)	0.25443 (13)	0.0578 (5)	
H14	0.3579	0.6417	0.1929	0.069*	
C15	0.40929 (10)	0.57473 (13)	0.28425 (13)	0.0539 (5)	
H15	0.4204	0.5345	0.2435	0.065*	
C16	0.43038 (9)	0.57010 (12)	0.37372 (12)	0.0474 (4)	
H16	0.4559	0.5263	0.3937	0.057*	
N3	0.35733 (9)	0.75046 (11)	0.46079 (11)	0.0581 (4)	
C17	0.33979 (10)	0.77941 (12)	0.52739 (14)	0.0531 (4)	
S1	0.31534 (3)	0.82304 (4)	0.61376 (4)	0.07149 (18)	
C18	0.73280 (8)	0.61190 (11)	0.64634 (11)	0.0422 (4)	
C19	0.76769 (9)	0.67895 (11)	0.62281 (12)	0.0456 (4)	
C20	0.83405 (10)	0.67559 (14)	0.59531 (14)	0.0564 (5)	
H20	0.8569	0.7217	0.5787	0.068*	
C21	0.86599 (10)	0.60474 (15)	0.59256 (15)	0.0617 (5)	
H21	0.9114	0.6020	0.5748	0.074*	
C22	0.83267 (11)	0.53773 (14)	0.61539 (16)	0.0629 (5)	
H22	0.8551	0.4890	0.6131	0.075*	
C23	0.76637 (10)	0.54120 (12)	0.64176 (14)	0.0529 (4)	
H23	0.7436	0.4946	0.6569	0.063*	
N4	0.73517 (9)	0.75053 (11)	0.62762 (13)	0.0587 (4)	
C24	0.72587 (9)	0.81716 (12)	0.63222 (14)	0.0510 (4)	
S2	0.71060 (3)	0.90666 (3)	0.63860 (5)	0.07015 (18)	
O1	0.5000	0.76596 (11)	0.7500	0.0511 (4)	
C31	0.54792 (10)	0.81107 (12)	0.70173 (15)	0.0555 (5)	
H31A	0.5892	0.7798	0.6939	0.067*	
H31B	0.5604	0.8578	0.7374	0.067*	
C32	0.52032 (14)	0.83590 (17)	0.61075 (17)	0.0761 (7)	
H32A	0.5068	0.7898	0.5759	0.114*	
H32B	0.5550	0.8644	0.5788	0.114*	
H32C	0.4812	0.8697	0.6184	0.114*	
O2	0.9968 (9)	0.5749 (3)	0.7693 (6)	0.092 (3)	0.5
C41	1.0756 (8)	0.5710 (9)	0.6491 (11)	0.155 (6)	0.5
H41A	1.0997	0.6007	0.6059	0.233*	0.5
H41B	1.1070	0.5431	0.6873	0.233*	0.5
H41C	1.0464	0.5346	0.6182	0.233*	0.5
C42	1.0345 (7)	0.6237 (9)	0.7061 (9)	0.114 (4)	0.5
H42A	1.0636	0.6605	0.7364	0.136*	0.5
H42B	1.0035	0.6521	0.6679	0.136*	0.5
C43	0.9492 (8)	0.6115 (8)	0.8328 (10)	0.124 (5)	0.5
H43A	0.9091	0.6246	0.7988	0.148*	0.5
H43B	0.9660	0.6586	0.8600	0.148*	0.5
C44	0.9249 (6)	0.5580 (6)	0.8994 (8)	0.117 (3)	0.5
H44A	0.8920	0.5822	0.9363	0.176*	0.5
H44B	0.9064	0.5111	0.8736	0.176*	0.5
H44C	0.9642	0.5455	0.9356	0.176*	0.5

### Atomic displacement parameters (Å^2^)

**Table:**

	*U*^11^	*U*^22^	*U*^33^	*U*^12^	*U*^13^	*U*^23^
Zn1	0.03477 (15)	0.04689 (17)	0.03351 (15)	0.000	0.00291 (10)	0.000
N1	0.0356 (7)	0.0494 (8)	0.0350 (7)	−0.0013 (6)	0.0024 (5)	0.0005 (6)
N2	0.0361 (7)	0.0477 (8)	0.0339 (7)	0.0002 (6)	0.0028 (5)	0.0001 (5)
C1	0.0360 (8)	0.0453 (9)	0.0404 (8)	−0.0008 (6)	0.0033 (6)	0.0012 (7)
C2	0.0384 (8)	0.0556 (10)	0.0440 (9)	−0.0045 (7)	0.0012 (7)	−0.0001 (8)
C3	0.0407 (9)	0.0590 (11)	0.0394 (8)	−0.0046 (7)	−0.0009 (7)	−0.0013 (7)
C4	0.0387 (8)	0.0468 (9)	0.0364 (8)	−0.0011 (7)	−0.0008 (6)	−0.0003 (7)
C5	0.0408 (8)	0.0458 (9)	0.0359 (8)	−0.0009 (7)	0.0016 (6)	−0.0009 (6)
C6	0.0419 (8)	0.0465 (9)	0.0332 (8)	−0.0009 (7)	0.0032 (6)	0.0005 (6)
C7	0.0429 (9)	0.0639 (11)	0.0350 (8)	−0.0039 (8)	0.0049 (7)	0.0007 (7)
C8	0.0408 (9)	0.0634 (11)	0.0382 (9)	−0.0031 (8)	0.0074 (7)	−0.0001 (8)
C9	0.0384 (8)	0.0464 (9)	0.0377 (8)	−0.0012 (7)	0.0066 (6)	−0.0003 (7)
C10	0.0380 (8)	0.0440 (8)	0.0401 (8)	−0.0010 (6)	0.0052 (6)	−0.0005 (7)
C11	0.0395 (8)	0.0507 (10)	0.0365 (8)	−0.0032 (7)	0.0013 (6)	−0.0006 (7)
C12	0.0472 (9)	0.0573 (11)	0.0393 (8)	0.0030 (8)	0.0031 (7)	−0.0016 (7)
C13	0.0524 (11)	0.0778 (14)	0.0431 (10)	0.0109 (10)	−0.0023 (8)	0.0054 (9)
C14	0.0482 (10)	0.0903 (16)	0.0346 (9)	0.0001 (10)	−0.0016 (7)	−0.0027 (9)
C15	0.0495 (10)	0.0705 (13)	0.0418 (9)	−0.0087 (9)	0.0040 (7)	−0.0132 (9)
C16	0.0465 (9)	0.0516 (10)	0.0443 (9)	−0.0033 (7)	0.0032 (7)	−0.0037 (7)
N3	0.0655 (10)	0.0604 (10)	0.0485 (9)	0.0124 (8)	0.0023 (7)	−0.0006 (8)
C17	0.0506 (10)	0.0545 (11)	0.0540 (11)	0.0066 (8)	−0.0021 (8)	0.0003 (9)
S1	0.0758 (4)	0.0729 (4)	0.0663 (4)	0.0059 (3)	0.0115 (3)	−0.0197 (3)
C18	0.0377 (8)	0.0522 (9)	0.0368 (8)	−0.0008 (7)	0.0036 (6)	−0.0005 (7)
C19	0.0423 (9)	0.0526 (10)	0.0417 (8)	−0.0045 (7)	−0.0004 (7)	−0.0002 (7)
C20	0.0430 (9)	0.0734 (13)	0.0529 (10)	−0.0128 (9)	0.0049 (8)	0.0062 (9)
C21	0.0389 (9)	0.0876 (16)	0.0592 (12)	0.0013 (10)	0.0104 (8)	0.0032 (11)
C22	0.0483 (10)	0.0702 (14)	0.0707 (13)	0.0137 (10)	0.0129 (9)	0.0031 (11)
C23	0.0463 (10)	0.0548 (11)	0.0579 (11)	0.0035 (8)	0.0105 (8)	0.0038 (8)
N4	0.0569 (10)	0.0526 (10)	0.0665 (11)	−0.0068 (8)	0.0004 (8)	0.0012 (8)
C24	0.0418 (9)	0.0569 (12)	0.0543 (10)	−0.0058 (8)	0.0004 (7)	0.0018 (8)
S2	0.0700 (4)	0.0529 (3)	0.0870 (4)	0.0033 (2)	−0.0071 (3)	−0.0033 (3)
O1	0.0499 (10)	0.0455 (10)	0.0586 (11)	0.000	0.0160 (8)	0.000
C31	0.0510 (10)	0.0541 (11)	0.0620 (12)	−0.0087 (8)	0.0112 (9)	0.0022 (9)
C32	0.0862 (18)	0.0802 (16)	0.0623 (14)	−0.0162 (14)	0.0082 (12)	0.0123 (12)
O2	0.073 (3)	0.087 (2)	0.116 (8)	0.005 (3)	−0.004 (7)	−0.006 (3)
C41	0.152 (10)	0.135 (10)	0.179 (13)	0.050 (8)	−0.008 (10)	−0.063 (9)
C42	0.091 (7)	0.125 (8)	0.122 (9)	−0.016 (5)	−0.039 (6)	0.025 (7)
C43	0.125 (11)	0.099 (8)	0.145 (13)	0.046 (7)	−0.036 (9)	−0.035 (8)
C44	0.108 (6)	0.071 (5)	0.175 (11)	−0.013 (4)	0.030 (7)	−0.002 (6)

### Geometric parameters (Å, º)

**Table:**

Zn1—N2	2.0622 (13)	C20—C21	1.374 (3)
Zn1—N2^i^	2.0622 (13)	C20—H20	0.9500
Zn1—N1^i^	2.0684 (14)	C21—C22	1.376 (3)
Zn1—N1	2.0685 (14)	C21—H21	0.9500
Zn1—O1	2.1352 (19)	C22—C23	1.387 (3)
N1—C4	1.368 (2)	C22—H22	0.9500
N1—C1	1.370 (2)	C23—H23	0.9500
N2—C9	1.368 (2)	N4—C24	1.162 (3)
N2—C6	1.370 (2)	C24—S2	1.571 (2)
C1—C10^i^	1.397 (2)	O1—C31	1.436 (2)
C1—C2	1.443 (2)	O1—C31^i^	1.436 (2)
C2—C3	1.348 (2)	C31—C32	1.507 (3)
C2—H2	0.9500	C31—H31A	0.9900
C3—C4	1.440 (2)	C31—H31B	0.9900
C3—H3	0.9500	C32—H32A	0.9800
C4—C5	1.401 (2)	C32—H32B	0.9800
C5—C6	1.404 (2)	C32—H32C	0.9800
C5—C11	1.494 (2)	O2—C42^ii^	1.11 (2)
C6—C7	1.440 (2)	O2—C42	1.479 (12)
C7—C8	1.355 (3)	O2—C43	1.495 (11)
C7—H7	0.9500	O2—C41^ii^	1.912 (18)
C8—C9	1.440 (2)	C41—C44^ii^	0.755 (17)
C8—H8	0.9500	C41—C43^ii^	0.900 (18)
C9—C10	1.401 (2)	C41—C42	1.499 (14)
C10—C1^i^	1.397 (2)	C41—O2^ii^	1.912 (18)
C10—C18	1.494 (2)	C41—H41A	0.9600
C11—C16	1.391 (3)	C41—H41B	0.9599
C11—C12	1.398 (3)	C41—H41C	0.9600
C12—C13	1.390 (3)	C42—C43^ii^	0.703 (16)
C12—N3	1.393 (2)	C42—O2^ii^	1.11 (2)
C13—C14	1.370 (3)	C42—C42^ii^	1.92 (3)
C13—H13	0.9500	C42—H42A	0.9601
C14—C15	1.384 (3)	C42—H42B	0.9599
C14—H14	0.9500	C43—C42^ii^	0.703 (16)
C15—C16	1.385 (3)	C43—C41^ii^	0.900 (18)
C15—H15	0.9500	C43—C44	1.446 (14)
C16—H16	0.9500	C43—H43A	0.9599
N3—C17	1.170 (3)	C43—H43B	0.9600
C17—S1	1.576 (2)	C44—C41^ii^	0.755 (17)
C18—C23	1.389 (3)	C44—H44A	0.9600
C18—C19	1.394 (3)	C44—H44B	0.9600
C19—N4	1.392 (3)	C44—H44C	0.9599
C19—C20	1.394 (3)		
			
N2—Zn1—N2^i^	164.44 (8)	C31—O1—Zn1	122.65 (11)
N2—Zn1—N1^i^	88.85 (6)	C31^i^—O1—Zn1	122.65 (11)
N2^i^—Zn1—N1^i^	88.54 (6)	O1—C31—C32	111.82 (17)
N2—Zn1—N1	88.54 (6)	O1—C31—H31A	109.3
N2^i^—Zn1—N1	88.85 (6)	C32—C31—H31A	109.3
N1^i^—Zn1—N1	160.61 (8)	O1—C31—H31B	109.3
N2—Zn1—O1	97.78 (4)	C32—C31—H31B	109.3
N2^i^—Zn1—O1	97.78 (4)	H31A—C31—H31B	107.9
N1^i^—Zn1—O1	99.69 (4)	C31—C32—H32A	109.5
N1—Zn1—O1	99.69 (4)	C31—C32—H32B	109.5
C4—N1—C1	106.50 (14)	H32A—C32—H32B	109.5
C4—N1—Zn1	126.59 (11)	C31—C32—H32C	109.5
C1—N1—Zn1	126.82 (11)	H32A—C32—H32C	109.5
C9—N2—C6	106.88 (13)	H32B—C32—H32C	109.5
C9—N2—Zn1	126.26 (11)	C42^ii^—O2—C42	94.7 (13)
C6—N2—Zn1	126.16 (11)	C42^ii^—O2—C43	26.4 (9)
N1—C1—C10^i^	125.29 (15)	C42—O2—C43	120.3 (9)
N1—C1—C2	109.66 (14)	C42^ii^—O2—C41^ii^	51.6 (8)
C10^i^—C1—C2	125.05 (16)	C42—O2—C41^ii^	146.0 (10)
C3—C2—C1	106.91 (15)	C43—O2—C41^ii^	27.3 (8)
C3—C2—H2	126.5	C44^ii^—C41—C43^ii^	122 (3)
C1—C2—H2	126.5	C44^ii^—C41—C42	137 (3)
C2—C3—C4	107.20 (15)	C43^ii^—C41—C42	18.1 (13)
C2—C3—H3	126.4	C44^ii^—C41—O2^ii^	129 (2)
C4—C3—H3	126.4	C43^ii^—C41—O2^ii^	49.6 (12)
N1—C4—C5	125.53 (15)	C42—C41—O2^ii^	35.6 (7)
N1—C4—C3	109.70 (14)	C44^ii^—C41—H41A	60.7
C5—C4—C3	124.76 (16)	C43^ii^—C41—H41A	94.4
C4—C5—C6	125.25 (16)	C42—C41—H41A	110.3
C4—C5—C11	117.74 (15)	O2^ii^—C41—H41A	143.7
C6—C5—C11	116.99 (15)	C44^ii^—C41—H41B	114.2
N2—C6—C5	125.30 (15)	C43^ii^—C41—H41B	124.1
N2—C6—C7	109.32 (15)	C42—C41—H41B	108.7
C5—C6—C7	125.28 (16)	O2^ii^—C41—H41B	97.5
C8—C7—C6	107.28 (16)	H41A—C41—H41B	109.5
C8—C7—H7	126.4	C44^ii^—C41—H41C	50.2
C6—C7—H7	126.4	C43^ii^—C41—H41C	108.5
C7—C8—C9	106.85 (15)	C42—C41—H41C	109.4
C7—C8—H8	126.6	O2^ii^—C41—H41C	82.6
C9—C8—H8	126.6	H41A—C41—H41C	109.5
N2—C9—C10	125.61 (15)	H41B—C41—H41C	109.5
N2—C9—C8	109.64 (15)	C43^ii^—C42—O2^ii^	109 (2)
C10—C9—C8	124.73 (16)	C43^ii^—C42—O2	127 (2)
C1^i^—C10—C9	125.76 (16)	O2^ii^—C42—O2	20.8 (8)
C1^i^—C10—C18	116.90 (15)	C43^ii^—C42—C41	23 (2)
C9—C10—C18	117.33 (15)	O2^ii^—C42—C41	92.9 (12)
C16—C11—C12	117.55 (16)	O2—C42—C41	108.1 (13)
C16—C11—C5	120.98 (17)	C43^ii^—C42—C42^ii^	156 (3)
C12—C11—C5	121.45 (16)	O2^ii^—C42—C42^ii^	50.1 (8)
C13—C12—N3	117.92 (18)	O2—C42—C42^ii^	35.3 (7)
C13—C12—C11	121.16 (18)	C41—C42—C42^ii^	142.7 (10)
N3—C12—C11	120.92 (16)	C43^ii^—C42—H42A	107.4
C14—C13—C12	119.9 (2)	O2^ii^—C42—H42A	132.6
C14—C13—H13	120.0	O2—C42—H42A	112.6
C12—C13—H13	120.0	C41—C42—H42A	109.5
C13—C14—C15	120.15 (18)	C42^ii^—C42—H42A	96.5
C13—C14—H14	119.9	C43^ii^—C42—H42B	88.1
C15—C14—H14	119.9	O2^ii^—C42—H42B	102.7
C14—C15—C16	119.83 (18)	O2—C42—H42B	109.6
C14—C15—H15	120.1	C41—C42—H42B	108.8
C16—C15—H15	120.1	C42^ii^—C42—H42B	86.7
C15—C16—C11	121.34 (19)	H42A—C42—H42B	108.1
C15—C16—H16	119.3	C42^ii^—C43—C41^ii^	138 (3)
C11—C16—H16	119.3	C42^ii^—C43—C44	158 (2)
C17—N3—C12	157.6 (2)	C41^ii^—C43—C44	26.4 (16)
N3—C17—S1	176.6 (2)	C42^ii^—C43—O2	44.9 (18)
C23—C18—C19	117.82 (16)	C41^ii^—C43—O2	103.2 (17)
C23—C18—C10	121.48 (16)	C44—C43—O2	113.4 (10)
C19—C18—C10	120.68 (17)	C42^ii^—C43—H43A	83.7
N4—C19—C20	119.79 (18)	C41^ii^—C43—H43A	83.3
N4—C19—C18	118.78 (16)	C44—C43—H43A	102.7
C20—C19—C18	121.44 (19)	O2—C43—H43A	107.3
C21—C20—C19	119.2 (2)	C42^ii^—C43—H43B	86.1
C21—C20—H20	120.4	C41^ii^—C43—H43B	135.5
C19—C20—H20	120.4	C44—C43—H43B	111.5
C20—C21—C22	120.49 (18)	O2—C43—H43B	113.6
C20—C21—H21	119.8	H43A—C43—H43B	107.3
C22—C21—H21	119.8	C41^ii^—C44—C43	32.0 (18)
C21—C22—C23	120.1 (2)	C41^ii^—C44—H44A	115.3
C21—C22—H22	119.9	C43—C44—H44A	111.3
C23—C22—H22	119.9	C41^ii^—C44—H44B	82.6
C22—C23—C18	120.90 (19)	C43—C44—H44B	113.0
C22—C23—H23	119.5	H44A—C44—H44B	109.5
C18—C23—H23	119.5	C41^ii^—C44—H44C	126.1
C24—N4—C19	161.5 (2)	C43—C44—H44C	104.0
N4—C24—S2	178.01 (19)	H44A—C44—H44C	109.5
C31—O1—C31^i^	114.7 (2)	H44B—C44—H44C	109.5

Symmetry codes: (i) −*x*+1, *y*, −*z*+3/2; (ii) −*x*+2, *y*, −*z*+3/2.
